# Mental Health and Psychological Impact on Students with or without Hearing Loss during the Recurrence of the COVID-19 Pandemic in China

**DOI:** 10.3390/ijerph18041421

**Published:** 2021-02-03

**Authors:** Ying Yang, Yanan Xiao, Yulu Liu, Qiong Li, Changshuo Shan, Shulin Chang, Philip H.-S. Jen

**Affiliations:** 1 Department of Hearing and Speech Rehabilitation, Binzhou Medical University, Yantai 264003, China; 2020091005@bzmc.edu.cn (Y.X.); 2020091001@bzmc.edu.cn (Y.L.); 2019091002@bzmc.edu.cn (Q.L.); 184240107@bzmc.edu.cn (C.S.); 184240134@bzmc.edu.cn (S.C.); 2 Division of Biological Sciences and Interdisciplinary Neuroscience Program, University of Missouri-Columbia, Missouri, MO 65211, USA

**Keywords:** COVID-19, hearing loss, mental health, psychological impact

## Abstract

Background: This study compares the mental health and psychological response of students with or without hearing loss during the recurrence of the COVID-19 pandemic in Beijing, the capital of China. It explores the relevant factors affecting mental health and provides evidence-driven strategies to reduce adverse psychological impacts during the COVID-19 pandemic. Methods: We used the Chinese version of depression, anxiety, and stress scale 21 (DASS-21) to assess the mental health and the impact of events scale—revised (IES-R) to assess the COVID-19 psychological impact. Results: The students with hearing loss are frustrated with their disability and particularly vulnerable to stress symptoms, but they are highly endurable in mitigating this negative impact on coping with their well-being and responsibilities. They are also more resilient psychologically but less resistant mentally to the pandemic impacts than the students with normal hearing. Their mental and psychological response to the pandemic is associated with more related factors and variables than that of the students with normal hearing is. Conclusions: To safeguard the welfare of society, timely information on the pandemic, essential services for communication disorders, additional assistance and support in mental counseling should be provided to the vulnerable persons with hearing loss that are more susceptible to a public health emergency.

## 1. Introduction

In December 2019, a serious public health emergency induced by coronavirus broke out in Wuhan, Hubei Province, China [1]. On 30 January 2020, the World Health Organization declared the coronavirus pandemic as a public health emergency of international concern and named the virus-induced disease COVID-19 on 11 February 2020. Similar to other RNA viruses, this virus has a significant genetic variation with a high recombination rate, which makes it disseminate easily in humans and animals around the world [2].

This is another public health emergency experienced by humans along with those in the past decade, including the SARS-CoV pandemic in 2003, Ebola in Sierra Leone in 2014, MERS-CoV in South Korea in 2015, and Ebola in the Democratic Republic of the Congo in 2018. This series of public health emergencies has inflicted varying degrees of psychological trauma and mental disorder on the general population [3,4,5].

Although the pandemic in China has been under control and entered a phase of normalization since 29 April 2020, there have still been sporadic new COVID-19 cases numbering less than 100 since 14 May 2020. From 11 June 2020, there has been a sudden recurrence of new confirmed and suspected COVID-19 cases in Beijing, the capital of China (National Health Commission of the People’s Republic of China). This pandemic recurrence has produced new alarm and concern about COVID-19 among the general public. According to the global dashboard of the World Health Organization, as of 9 January 2021, there have been 87, 589, 206 infected patients and 1, 906, 606 deaths caused by the COVID-19 pandemic [6].

When the epidemic becomes a pandemic as a public health emergency of global concern, it requires mutual learning and cooperation among all countries to maintain global health [7]. A previous study has shown that home quarantine is more effective than traffic restrictions in the control of population movement and the spread of the virus [8]. When people are under home quarantine or isolation, they inevitably encounter a shortage of daily necessities, the restriction of daily travel, the loss of income, and a high degree of uncertainty resulting in varying degrees of paranoiac concerns [9,10,11]. All these sudden negative impacts on their lifestyle have led to the emergence and development of a series of mental health problems and unbearable psychological impacts [12].

Since its outbreak, the worldwide spread of the COVID-19 has produced a series of mental health problems. Wang. et al. found that among the general population in China, more than half of the respondents rated the psychological impact as moderate to severe [10]. Lai et al. investigated relevant medical staff during the COVID-19 outbreak in China and discovered that a considerable number of them had symptoms such as depression, anxiety, insomnia, and distress [13]. Cao reported that about 24.9% of college students have experienced anxiety because of this COVID-19 outbreak [14]. However, there is currently no specific research on the mental health of students with hearing loss during the pandemic.

According to Luey et al., the mental health of those with hearing loss was worse than that of the general population [15]. Previous studies have shown that deafness is associated with large heterogeneity in cognitive, social, and emotional development [16]. The psychological studies related to COVID-19 or SARS mostly focused on the psychological status of ordinary people during the pandemic; mental health counseling may also be needed for people with hearing loss during the COVID-19 pandemic [17,18]. A previous study has shown that congenital sensorineural hearing loss can delay the development of speech, language, and social communication skills [19]. Compared with their peers, the unique social and emotional development patterns of persons with hearing loss may make them more susceptible to psychological and emotional distress with lower quality of life due to ineffective communication tools and services during the pandemic [20,21,22,23]. Owing to the limitation of their spoken and written language level, people with hearing loss might find it more difficult to communicate effectively when compared with those with normal hearing and their psychological demands might be more difficult satisfy.

Investigators identified a four-fold increase in symptoms of anxiety and depression in those with hearing loss when compared with the general population [24]. Compared with healthy populations, deaf people are at a higher risk of mental health problems. A study has indicated that psychiatric patients suffered from more severe psychiatric symptoms during the COVID-19 with higher scores for a range of symptoms including anxiety, depression, stress, post-traumatic stress disorder, insomnia, poor physical health, anger, impulsivity, and higher suicidal ideations [25]. Moreover, information and resources regarding COVID-19 are not always easily accessible for deaf people [26]. Therefore, the COVID-19 pandemic may be likely to create or exaggerate mental health problems in the hearing loss group. However, there was rarely research to explore the psychological impact of COVID-19 on people with hearing loss.

For all these reasons, we hypothesize that the degree of COVID-19-induced mental health and psychological impact would be greater for persons with than without hearing loss. To test this hypothesis, we examine the relevant factors that contribute to the difference in the impact of COVID-19 on the mental health status and psychological response between these two groups of persons during the recurrence of the COVID-19 pandemic in Beijing, the capital of China.

## 2. Materials and Methods

### 2.1. Study Design and Study Population

We conducted our survey from 15 to 27 June, when there was a sudden recurrence of new COVID-19 cases from 11 June, in Beijing, the capital of China. Using the cross-sectional survey design and snowball sampling method, we anonymously collected the relevant data on the immediate psychological responses of students with or without hearing loss from junior and high schools and universities using questionnaire star, an online questionnaire survey platform. During the data collection period, none of the students with or without hearing loss had officially returned to school; the study advert is a QR code randomly generated by questionnaire star, posters of questionnaires were sent to teachers in higher education institutions and special education schools and the questionnaire was transmitted through WeChat or Tencent QQ. Around 120 college students with hearing loss, 300 senior and junior high school students with hearing loss, 500 college students with normal hearing, and 300 senior and junior high school students with normal hearing were invited. The individuals with permanent hearing loss were enrolled in the survey; they came from the universities (Binzhou Medical University, Shandong, China, Changchun University, Jilin, China, etc.) and special education schools (Yantai, Qingdao, Beijing, Harbin, etc.) that could provide educational services for people with disabilities. In addition to their participation, a respondent was encouraged to invite new respondents from his or her contacts. Although the questionnaire was filled out anonymously, many students were reluctant to take the survey.

### 2.2. Study Tool

The survey questionnaires collected information from the respondents in three categories. (1) Demographic data including gender, age, educational background, parents’ profession and education level, family living status, systemic disease status, communication means, satisfaction with current communication mode, bedtime, and physical activity (Table 1); (2) the precautionary measures, concerns, and availability of public service including avoiding sharing of utensils (e.g., chopsticks) during mealtime, washing hands with soap and water, washing hands immediately after coughing, or sneezing, rubbing the nose, and wearing a mask with or without the pandemic symptoms, the daily average number of hours staying at home, testing for COVID-19, concern about their family members becoming infected during the COVID-19, pandemic recurrence in Beijing and the worldwide pandemic, needing help and mental health counseling, impact on lifestyle, availability of educational support, special assistance from the school, community and government (Table 2); and (3) information on hearing loss from students with hearing loss (SHL) including the family history of deafness, hereditary connection, cause of deafness, degree of hearing loss, and the use of hearing aids or devices.

We used the Chinese version of depression, anxiety, and stress scale 21 (DASS-21) to evaluate the mental health of the respondents. This DASS-21 has been proven to be a reliable and effective measure [27]. The depression subscale consists of items 3, 5, 10, 13, 16, 17, and 21 with scores ranging from normal (0–9), mild (10–13), moderate (14–20), severe (21–27) to extremely severe (28–42). The anxiety subscale consists of items 2, 4, 7, 9, 15, 19, and 20, with scores ranging from normal (0–7), mild (8–9), moderate (10–14), severe (15–19), to extremely severe (20–42). Finally, the stress subscale consists of items 1, 6, 8, 11, 12, 14, and 18, with scores ranging from normal (0–14), mild (15–18), moderate (19–25), severe (26–34) to extremely severe (35–42).

The Impact of Events Scale—Revised (IES-R) is a self-reporting tool widely used in the field of psychological impact and has been proven to be suitable for the Chinese population [28]. The scale includes 22 items with a score of 0–4 in five grades, and the total score is 0–88. The scale of the psychological impact ranges from normal (0–23), mild (24–32), moderate (33–36) to severe (≥37).

To determine the association between the psychological inflexibility of students with hearing loss and their mental and psychological responses to the pandemic, we designed a survey questionnaire (Appendix A) using a previous study which developed an Acceptance and Action Questionnaire—Adult Hearing Loss (AAQ-AHL) as a reference [29]. It mainly provides feedback on three aspects related to hearing loss, including cognitive feedback, negative thoughts and feelings, and behavioral avoidance aspects of psychological inflexibility. The questionnaire survey includes 12 questions relating to their frustration and negative feeling of hearing loss. These items are rated from 0 (never true) to 4 (always true). Items 1, 2, 5, and 6 are reverse-scored. Individual item scores are summed up to create a total score. Higher scores reflect higher psychological inflexibility.

### 2.3. Statistical Analysis

We obtained the percentage of responses to each categorical variable by dividing the number of responses to each categorical variable by the total number of responses. We used the Chi-squared test to analyze the differences in categorical variables between the two groups of students. We then analyzed the differences in the degree of DASS-stress, anxiety, depression, and psychological impact using the independent sample *t*-test to compare the mean score between them. Furthermore, the above cut-off scores of DASS-21 stress (>14), anxiety (>7), depression (>9) subscales, and IES-R (>23) for the respondents were for psychological and post-traumatic stress disorder symptoms, we used binary logistic regressions to calculate the univariable association between independent and dependent variables for these two groups of students, respectively. Finally, internal consistency reliability, construct validity, and criterion validity were used to evaluate the psychometric properties of the AAQ-AHL. Total scores of AAQ-AHL were included in binary logistic regressions models for further investigation. All statistical tests were two-tailed with a significance level of *p* < 0.001–0.05 using SPSS Statistic 21.0 (IBM Corporation, New York, NY, USA).

## 3. Results

### 3.1. Comparison of Mental Health Status and Psychological Impact between Students with or without Hearing Loss

There were 544 students with normal hearing (hereafter called SNH) from 94 cities, and 174 students with hearing loss (hereafter called SHL) from 35 cities in China responding to our survey but only 542 (99.63%) SNH and 164 (94.25%) SHL completed the survey questionnaire. Therefore, only data collected from these 706 students were used in this study.

To determine the possible difference in pandemic-induced mental and psychological impacts between SNH and SHL, we compared their mean scores and rate of occurrence of DASS-21 and IES-R scores.

The means and standard deviations of DASS-21 stress, anxiety, depression subscales, and IES-R scores were 7.24 ± 8.09, 4.16 ± 6.44, 3.93 ± 7.07 and 13.35 ± 15.31 for SNH, and 8.56 ± 8.26, 3.84 ± 5.80, 4.43 ± 7.32 and 11.00 ± 13.72 for SHL, respectively. The mean score for DASS stress and depression subscales is slightly higher for the SHL than for the SNH. Conversely, the mean score for the DASS anxiety subscale and IES-R is slightly higher for the SNH than for the SHL. However, all these differences are statistically insignificant (Student’s *t*-test. *p* ≥ 0.05).

The numbers of students with mild to extremely severe DASS-21 stress, anxiety, depression subscales and IES-R were 72 (13%), 116 (21%), 83 (15%) and 129 (24%) for SNH, and 61 (37%), 36 (22%), 31 (19%) and 23 (14%) for SHL, respectively. The rate of occurrence is higher in all three DASS-21 subscales but is lower in IES-R for the SHL than for the SNH. A statistical analysis shows a significant difference between the two groups in the DASS-21 stress subscale (χ^2^ = 47.08, *p* < 0.001) and IES-R (χ^2^ = 7.12, *p* < 0.01) but not in the DASS-21 anxiety (χ^2^ = 0.02, *p* > 0.05) and depression (χ^2^ = 1.20, *p* > 0.05) subscales.

### 3.2. Association of Demographic Variables with Mental Status and Psychological Impact

As shown in Table 1, the chi-square test shows a significant difference between SNH and SHL in gender (χ^2^ = 5.57, *p* < 0.05) and age (χ^2^ = 242.31, *p* < 0.001). Our study shows that male SNH (37.27%) displayed a significantly higher degree of depression scores than female SNH (odds ratio (OR) = 2.11, 95% CI: 1.36 to 3.29, *p* < 0.01). Moreover, the SNH at the age of 13–18 years old (7.2%) had a higher degree of stress (OR = 5.81, 95% CI: 1.35 to 25.05, *p* < 0.01), anxiety (OR = 7.77, 95% CI: 1.84 to 32.85, *p* < 0.01), depression (OR = 9.09, 95% CI: 2.35 to 35.13, *p* < 0.01) and IES-R (OR = 14.30, 95% CI: 3.75 to 54.53, *p* < 0.001) scores than the age group of 19–27 years old (92.8%). However, SHL at the age of 13–18 years old (62.8%) had a significantly lower degree of stress score than the age group of 19–27 years old (OR = 0.41, 95% CI: 0.18 to 0.95, *p* < 0.05).

By the same token, the chi-square test shows a significant difference between SNH and SHL in father’s education level (χ^2^ = 31.98, *p* < 0.001), mother’s education level (χ^2^ = 21.87, *p* < 0.001), father’s profession (χ^2^ = 6.93, *p* < 0.05) and mother’s profession (χ^2^ = 11.25, *p* < 0.01). The SNH with the father’s education at senior high school and above (42.62%) show significantly higher stress scores (OR = 2.21, 95% CI: 1.09 to 4.47, *p* < 0.05), and the SNH with the mother’s education level at senior high school and above (33.58%) show a significantly lower level of anxiety (OR = 0.53, 95% CI: 0.28 to 0.98, *p* < 0.05) and depression (OR = 0.50, 95% CI: 0.28 to 0.90, *p* < 0.05) than at junior high school and levels below. However, SHL with the father’s education at senior high school and above (18.29%) display a significantly lower IES-R score (OR = 0.06, 95% CI: 0.003 to 0.90, *p* < 0.05). On the other hand, SHL whose fathers are farmers, workers or employed (64.02%) display a significantly lower stress score (OR = 0.22, 95% CI: 0.05 to 0.98, *p* < 0.05), and the SHL whose mothers are jobless or run an individual business (45.12%) have significantly higher anxiety scores (OR = 4.05, 95% CI: 1.16 to 14.22, *p* < 0.05).

Based on the chi-square test, there is a significant difference between SNH and SHL in household mode (χ^2^ = 37.57, *p* < 0.001), communicative means (χ^2^ = 592.64, *p* < 0.001) and satisfaction with current communication mode (χ^2^ = 87.28, *p* < 0.001). SHL living with other or grandparents (10.37%) show a higher depression level than those with parents (58.54%) during the pandemic (OR = 5.39, 95% CI: 1.27 to 22.81, *p* < 0.05). Those SNH with satisfaction with their current communication mode (78.97%) show a lower level of stress (OR = 0.52, 95% CI: 0.29 to 0.96, *p* < 0.05), anxiety (OR = 0.59, 95% CI: 0.35 to 0.97, *p* < 0.05), and depression score (OR = 0.59, 95% CI: 0.36 to 0.95, *p* < 0.05) than those recording fair and dissatisfied. Meanwhile, SHL using gesture language or both gesture and lip-language or using written word (87.20%) for communication show a significantly lower stress score than those using mandarin for communication (OR = 0.23, 95% CI: 0.06 to 0.85, *p* < 0.05). Furthermore, SHL with the satisfaction with their current communication mode (40.85%) display a lower stress score than those recording fair and dissatisfied (OR = 0.20, 95% CI: 0.08 to 0.49, *p* < 0.001).

The chi-square test shows a significant difference between SNH and SHL in bedtime (χ^2^ = 199.31, *p* < 0.001). Bedtime does not appear to play a role in affecting the mental health status and psychological impact of the SHL. On the other hand, those SNH with a bedtime before midnight show a significantly lower stress, anxiety, depression, and IES-R score than those SNH with a bedtime after midnight (6.64%), especially for those with a bedtime between 9:00–11:00 p.m. (64.03%).

### 3.3. Association of Precautionary Measures, Concerns, Public Service with Mental Health Status and Psychological Impact

Table 2 provides an overview of the relationship of precautionary measures, concerns, public service with mental health status, and psychological impact. Most students always or most of the time have practiced precautionary measures and display concerns about the pandemic. Those SHL who most of the time (19.51%) avoid sharing utensils during meals displayed a significantly higher stress score when compared with the SHL who always (32.93%) practiced such a precautionary measure (OR = 3.54, 95% CI: 1.21 to 10.35, *p* < 0.05). Similarly, those SHL who most of the time (14.02%) wear a mask regardless of the presence of symptoms displayed significantly higher stress (OR = 6.12, 95% CI: 1.73 to 21.66, *p* < 0.01) and depression scores (OR = 6.19, 95% CI: 1.17 to 32.66, *p* < 0.05) than those SHL who always (74.39%) wear masks. Conversely, those SHL who most of the time (16.46%) wash their hands with soap and water showed a significantly lower depression score when compared to those SHL who always (71.34%) wash their hands with soap and water (OR = 0.04, 95% CI: 0.003 to 0.36, *p* < 0.05). Meanwhile those SNH who sometimes or occasionally or never (10.52%) wash their hands with soap and water displayed a significantly higher anxiety score when compared with those SNH who always (69.37%) wash their hands with soap and water (OR = 2.34, 95% CI: 1.08 to 5.08, *p* < 0.05).

While the majority of them did not have COVID-19 testing (94.28% for SNH, 93.29% for SHL), most of them were not concerned about family members becoming infected during the COVID-19 outbreak in Beijing (73.8% for SNH, 62.2% for SHL). Nevertheless, they were concerned by a new COVID-19 outbreak in China and the outbreak of pandemic overseas (57% for SNH, 60.37% for SHL). For those SNH who were not concerned about a new COVID-19 outbreak in China during the recent confirmed cases of COVID-19 overseas, they displayed a lower stress score (OR = 0.53, 95% CI: 0.28 to 0.99, *p* < 0.05). While most of them found educational support from the school during the pandemic (90.41% for SNH, 73.78% for SHL), they failed to find community special assistance during the pandemic (57.56% for SNH, 64.02% for SHL). More than half of SNH (52.4%) thought that the local government had provided special attention and support during the pandemic. However, most (70.12%) of the SHL did not think so.

On the other hand, the majority of them neither needed help (90.59% for SNH, 90.24% for SHL) nor received mental health counseling during the pandemic (86.53% for SNH, 90.24% for SHL). For most SNH (90.59%) who did not need help during the pandemic, they displayed significantly lower IES-R (OR = 0.44, 95% CI: 0.23 to 0.84, *p* < 0.05) scores. For those most SHL (90.24%) who did not receive mental health counseling during the COVID-19 pandemic, they also displayed a significantly lower depression score (OR = 0.10, 95% CI: 0.02 to 0.52, *p* < 0.01).

The chi-square test shows a significant difference between SNH and SHL in the item of lifestyle affected by the COVID-19 pandemic (χ^2^ = 29.63, *p* < 0.001). About half (49.82%) of the SNH thought the pandemic did not affect their lifestyle and they displayed significantly lower stress (OR = 0.43, 95% CI: 0.24 to 0.77, *p* < 0.01), anxiety (OR = 0.62, 95% CI: 0.39 to 0.99, *p* < 0.05), depression (OR = 0.57, 95% CI: 0.37 to 0.88, *p* < 0.05) and IES-R (OR = 0.46, 95% CI: 0.29 to 0.73, *p* < 0.01) scores. Meanwhile, the most (73.78%) of the SHL did not think that their lifestyle was affected by the pandemic and thus displayed a significantly lower stress score (OR = 0.39, 95% CI: 0.17 to 0.92, *p* < 0.05).

### 3.4. Psychological Inflexibility and Its Association with Mental Health Status and Psychological Impact for SHL

According to our survey, the hearing loss of 164 SHL is due to congenital (33.54%), ototoxic drug (18.90%), inheritance (7.93%), virus infection (3.05%), trauma (1.22%), and other or unknown sources (35.36%). Most of them are not due to family history or hereditary connection (86.59%). Most (70.73%) of them have extremely severe hearing loss such that they (73.17%) either use hearing aids, cochlear implants, or both. The remaining SHL (26.83%) do not use any hearing device. We further explored the relation of psychological inflexibility with the mental health status and psychological impact of these 164 SHL. According to the AAQ-AHL, we found that most of the SHL who always and most of the time lead a full life (52.44%) believed that their lives were going well (59.76%), pursued their goals (44.51%) as well as wished to take care of their responsibilities (65.86%).

To gain more insight into the relationship between hearing loss and mental health status as well as psychological impact, we evaluated the psychometric properties of the AAQ-AHL, including internal consistency reliability, construct validity, and criterion validity.

The results of exploratory factor analysis showed that the Kaiser-Mayer-Olkin (KMO) value was 0.837, and the chi-square value of the Bartlett sphericity test was 1094.986 (df = 66, *p* < 0.001), which indicated that the sample data were suitable for exploratory factor analysis. The principal component analysis showed two factors with eigenvalues that exceeded unity and accounted for 63.72% of the total variance. The scree plot showed that two factors could be extracted. Factor one is related to behavioral avoidance caused by hearing loss, including items 3, 4, 7, 8, 9, 10, 11, and 12. Factor two is associated with the degree of acceptance of hearing loss, including items one, two, five and six. Internal consistency of factors one and two was excellent (α = 0.894 and 0.865, respectively). All items had high loadings on a single factor, ranging from 0.644 to 0.887. The total score of the AAQ-AHL was significantly correlated with the total score of DASS-21 (r = 0.285, *p* < 0.01).

The results above indicate that the scale is a useful measure for assessing psychological inflexibility among students with hearing loss. Binary logistic regression indicated that the total scores of the AAQ-AHL was significantly associated with the DASS stress score (OR = 1.09, 95%CI: 1.04 to 1.14, *p* < 0.001), rather than with anxiety (OR = 1.01, 95%CI: 0.97 to 1.06, *p* > 0.05), depression (OR = 1.00, 95%CI: 0.95 to 1.05, *p* > 0.05) and IES-R (OR = 1.05, 95%CI: 0.99 to 1.11, *p* > 0.05). Overall, we observed that SHL were frustrated with their disability and particularly vulnerable to stress symptoms.

## 4. Discussion

Although the varying degree of difference in the mean DASS-21 and IES-R scores between the SNH and SHL does not differ significantly, the overall mean IES-R score for both groups is lower than 24 points, suggesting the lack of post-traumatic stress disorder symptoms. This finding differs from a previous study conducted during the peak of the COVID-19 outbreak across China when the twice-collected mean IES-R scores of the general public were higher than 24 points [17]. This different observation between the two studies is likely because we conducted our study during the stabilization stage of pandemic prevention and control in China when the number of sporadic COVID-19 contracted cases was always below 100. For this reason, both groups of students might already have more comprehensive and assured knowledge about the pandemic. As a result, they do not show post-traumatic stress disorder symptoms during our survey period.

We found that the SHL have a significantly higher rate of occurrence in the DASS-21 stress subscale but a lower one in the IES-R than the SNH have. This finding suggests that SHL are less resistant mentally but more resilient psychologically to the COVID-19 pandemic impacts. Conceivably, the poor satisfaction of communication, special assistance, and attention provided by the community and local government experienced by the SHL would produce more mental issues induced by the pandemic (Table 1 and Table 2). On the other hand, their long-term life experience with disability in hearing may have strengthened their psychological resilience in dealing with the pandemic impact.

Previous studies have shown that, under the impact of public health emergencies, female subjects exhibit higher levels of stress, anxiety, and depression than male subjects do [10,30,31]. Different from these studies, we find that male SNH have a higher degree of anxiety and depression, and the SHL did not show significant differences in gender (Table 1). This finding is also different from a previous report that shows that both male and female students display similar stresses and negative emotions under the impact of the pandemic [14]. Meanwhile, prior studies on what happens to health care workers facing COVID-19 also indicated that females are likely to experience higher occupational stress and mental problems [32,33].

The SNH at a younger age (13–18 years) have a significantly higher degree of stress, anxiety, depression, and IES-R scores than the older age group (19–27 years). On the contrary, the SHL at a younger age have a significantly lower degree of stress than the older age group (Table 1). This age-dependent response to pandemic impact has been reported previously [34]. A study in Malaysia noted that young people were more likely to have negative reactions due to a larger deal of attention on social networks [35], and it found that social media makes it easier to access information, which may be essential during a lockdown, but the “always-on” facet of social media can be exhausting and potentially damaging to students’ mental health. The flow of risk-elevating messages on social media that are portrayed in a very negative manner could trigger anxiety [35]. We suspect that this may be due to hearing loss, which generally leads to language development retardation in children with hearing loss, and younger SHL may be less active in social networks so that they are not disturbed by rumors or misleading information. Therefore, the psychological impact of the COVID-19 pandemic in terms of the stress score is significantly lower for the SHL at 13–18 years than 19–27 years.

It is interesting to find the association of COVID-19-induced impacts with the education of the parents for SHL and SNH. It indicated that the fathers of SNH having a higher education level (senior higher school and above) was a dangerous factor against stress, but it was a protective factor against IES-R for SHL, and the mothers of SNH having a higher education level was a protective factor against anxiety and depression. While we are not sure about the significance of this dichotic finding, we do find that the fathers of the SNH have a higher percentage of education level above senior high school than the fathers of the SHL have (42.62% vs. 18.29%) and given the result in which fathers and mothers of SNH with higher education have completely differing performances, there is not a clear explanation for this phenomenon using prior studies as a reference. Furthermore, we observed that SHL who lived with others or grandparents had higher levels of anxiety than those who lived with their parents during the pandemic. Reviewed research highlights the importance of thinking beyond the level of individual children in a post-disaster environment, with the recognition of the potential role of constructs such as parents’ coping and mental health, parenting practices, and the broader family environment [36]. Based on these findings, future studies will be needed to refine and validate whether it is the mother or father who plays the primary versus secondary role in the psychology of both sets of students in the family.

On the other hand, pandemic-induced self-isolation and travel restrictions would reduce the workforce across all sectors of the economy and the scope of the job market [37]. As a result, the pandemic inevitably would lead to changes in parents’ work, increased family pressure and burden, and would produce an impact on the mental health of families [38]. We found that SHL with the mothers’ profession being jobless or individual business was associated with a higher level of anxiety. For all these reasons, we can conceivably speculate that mothers of the SHL would be more susceptible to various pandemic-induced impacts than the parents of the SNH would be. These pandemic-induced impacts on their parents would inevitably also affect the mental and psychological responses of the SHL to the pandemic.

Effective communication is the basis for timely access to pandemic information. We found that gesture language alone or together with lip-language or words is the most effective communication means for the SHL. This is substantiated by the finding that most SHL (87.2%) who used these three means for communication displayed a significantly lower stress score while those few SHL (12.80%) using Mandarin for communication during the pandemic.

Previous studies have shown that people with hearing loss encounter more problems in seeking health services due to the limitation of communication for the timely relief of psychological distress [23,39]. In agreement with these studies, we found that the SHL displayed a significantly lower stress score when they were satisfied with their current communication mode. This phenomenon can also be observed in SNH, with those satisfied with their current communication mode being associated with a lower level of stress, anxiety, and depression. We speculate that learning more information on the pandemic through a satisfactory communication mode might decline their degree of psychological response to the pandemic impact.

It is also interesting to find that bedtime plays an important role in COVID-19 induced mental and psychological impacts only for the SNH but not for the SHL who mostly (75.61%) go to bed before 10 pm. Those SNH with a bedtime before 11 p.m. have significantly lower DASS-21 and IES-R scores. Assuming both groups of students typically wake up before 7:30 a.m. for their schooling, all would have at least 8 h of sleep. Conceivably, this sufficient sleeping time would strengthen their health condition in response to the COVID-19-induced impacts.

When people with hearing disabilities can achieve successful communication, they must have as much visual information as possible, particularly those who rely on sign language or lip-reading [40]. Most masks prevent visual access to the speaker’s lips and create a barrier during communication, which could hinder speech perception especially when the listener has a hearing impairment [41]. Corey’s experiments also have shown that face masks can attenuate a speaker’s high-frequency sound, with the strongest attenuation above 4 kHz, which makes verbal communication more difficult [42]. Previous studies have shown that before the COVID-19 pandemic, the mask-wearing rates were 20 of 1745 individuals (1.1%) in mainland China, and the qualified mask-wearing rate in mainland China was the lowest among the regions [43]. The mask-wearing rate may be likely to be lower in the population with hearing loss before the COVID-19 pandemic. However, the rate of mask-wearing increased dramatically due to the need for protection during the pandemic. Our study unexpectedly found that the proportion of SHL who always wore masks (74.39%) was higher than that of SNH (66.79%). It suggested that SHL were able to insist on wearing masks even though masks may alter the intelligibility of speech communication. Our study also suggested that wearing a mask most of the time regardless of the presence or absence of symptoms compared to always, contributed to increasing stress and depression of SHL. What is similar is that avoiding sharing of utensils during meals most of the time as opposed to always tends to be associated with higher levels of stress. This might be explained by the suggestion that the less protective measures are adopted, the weaker the protective effects on psychology. This is also consistent with the research of Wang et al. [10].

An unexpected finding of the present study was that high adherence to the personal hygiene factor of washing hands with soap and water was predictive of higher levels of depression. One possible explanation is that SHL with depressive symptoms are more likely to take comprehensive precautions against infection due to the difficulty of identifying the truth of media reports on the disastrous effects of the COVID-19 pandemic. On the other hand, repeated emphasis on the need to adhere to protective measures may have contributed to the association between depression levels and high adherence to washing hands with soap and water. Further studies are needed to investigate these hypotheses.

Nevertheless, we found that those SNH who sought help during the pandemic displayed high a score in IES-R. These findings are in agreement with the report that the help-seeking behavior of patients is related to depressive symptoms [44]. It is interesting to note that most students in both groups (86.53% for SNH; 90.24% for SHL) did not need mental health counseling during the pandemic. Those few (9.76%) SHL who received mental health counseling displayed a higher depression score presumably due to their apprehension of the pandemic. It is also interesting to note that more (50.18%) SNH but fewer (26.22%) SHL believed that the COVID-19 pandemic has affected their lifestyle as shown by their higher scores for psychological effects. It is worth noting that the high scores of SNH were on the stress, anxiety, depression, and IES-R scales, while SHL only showed high scores for stress.

We found that that SHL are more susceptible to greater stress. We evaluated the degree of mental stiffness in students with hearing loss with an AAQ-AHL total score. A statistical association was found between stress and AAQ-AHL total score, but not in anxiety, depression, and IES-R. All these observations indicate that the psychological inflexibility of these SHL is not as rigid as our intuition suggests. While hearing loss may frustrate them in leading a full life activity and responsibilities, they are quite endurable in mitigating this negative impact on their coping with the pandemic impact. A longitudinal study, in China, surveyed the general population twice (during the initial outbreak and the pandemic’s peak four weeks later), finding that the pandemic has led to significantly higher post-traumatic stress disorder symptoms in both stages [17]. Meanwhile, very few studies have researched SHL’s mental level before or after the pandemic in China. Our study is not sure how much of the stress that we have observed is attributable to the pandemic. In addition, we also do not completely exclude the possibility that people with pre-existing mental health problems may have experienced worse outcomes during the pandemic. It is necessary to conduct a longitudinal study to examine the psychological indicators of SHL after the pandemic has been completely eradicated to clarify the answer.

Finally, it is important to indicate that our study has some limitations. Firstly, most of our respondents are SNH, and the number of SHL is relatively small. During the pandemic, isolation at home made it impossible for us to interview face to face; the filling-in of the questionnaire followed the principle of voluntariness. Due to the relatively large number of questions in our questionnaire, many SHL failed to complete the questionnaire. Future studies will expand the sample size of SHL. Secondly, this is not a longitudinal study and we did not collect the psychological indicators of students with hearing loss before the pandemic due to the unpredictability of the pandemic. We will conduct another investigation after the pandemic has completely passed to explore the psychological impact on SHL. Thirdly, our respondents are mainly students with or without hearing loss such that the observation may not apply to the population in all social strata. Fourthly, our respondents are in the age range between 13 and 27 years so our observation may not apply to the population across the age spectrum. Fifthly, self-reported levels of psychological impact and mental health status may not always be in line with objective assessment by mental health professionals. Finally, hearing loss may also contribute to the observed COVID-19-induced psychological impact and mental health status of these SHL. Another possibility for further research would be to re-investigate the psychological characteristics of the SHL in the post-pandemic period to clarify whether it is hearing loss itself or the combination of the pandemic and hearing loss that contributed to their abnormal psychological status to further determine the exact degree of psychological damage caused by the pandemic.

## 5. Conclusions

During the stabilization stage of the COVID-19 pandemic in China, all surveyed students did not show any significant increase in psychological impact but they displayed a high degree of stress when they felt that the pandemic had affected their lifestyle. Although the SHL are frustrated with their disability, they are highly endurable in mitigating this negative impact and are more resilient psychologically but less resistant mentally to the pandemic impacts than the SNH. The mental and psychological response to the pandemic is associated with more related factors and variables for the SHL than for the SNH.

To safeguard the welfare of the general population, the governments at all levels should disseminate timely information about the pandemic, ensure essential services, and support the public during the pandemic. In particular, additional assistance in mental counseling and communication should be provided to vulnerable persons with hearing loss that are more susceptible to the public health emergency during the pandemic.

## Figures and Tables

**Table 1:** Association of demographic variables with the mental health status and psychological impact for students with normal hearing (*n* = 542) and students with hearing loss (*n* = 164).

Demographic Variable	χ^2^	Students with Normal Hearing (*n* = 542)					Students with Hearing Loss (*n* = 164)				
		Percentage (*n*)	Stress	Anxiety	Depression	IES-R	Percentage (*n*)	Stress	Anxiety	Depression	IES-R
			OR (95% CI)	OR (95% CI)	OR (95% CI)	OR (95% CI)		OR (95% CI)	OR (95% CI)	OR (95% CI)	OR (95% CI)
Gender											
Male	5.57 *	37.27% (202)	1.52 (0.86~2.70)	2.36 (1.48~3.77)	2.11 ** (1.36~3.29)	1.01 (0.64~1.60)	47.56% (78)	1.34 (0.61~2.96)	0.64 (0.28~1.47)	0.39 (0.15~1.003)	1.20 (0.43~3.39)
Female		62.73% (340)	Reference	Reference	Reference	Reference	52.44% (86)	Reference	Reference	Reference	Reference
Age											
13~18 years old	242.31 ***	7.20% (39)	5.81 * (1.35~25.05)	7.77 ** (1.84~32.85)	9.09 ** (2.35~35.13)	14.30 *** (3.75~54.53)	62.80% (103)	0.41 * (0.18~0.95)	1.30 (0.52~3.24)	1.83 (0.65~5.15)	1.27 (0.39~4.12)
19~27 years old		92.80% (503)	Reference	Reference	Reference	Reference	37.20% (61)	Reference	Reference	Reference	Reference
Educational Background											
General school	589.12 ***	97.60% (529)	1.06 (0.18~6.13)	2.97 (0.52~16.82)	2.17 (0.47~10.04)	6.19 (0.68~55.95)	5.49% (9)	0.83 (0.15~4.58)	0.55 (0.05~5.98)	0.77 (0.05~12.34)	1.48 (0.10~21.57)
General and (or) special school		2.4% (13)	Reference	Reference	Reference	Reference	94.51% (155)	Reference	Reference	Reference	Reference
Current Education Level											
Junior or senior high school	351.23 ***	12.36% (67)	0.82 (0.23~2.91)	0.35 (0.10~1.25)	0.32 (0.10~1.05)	0.38 (0.12~1.20)	89.02% (146)	0.50 (0.13~1.87)	1.34 (0.29~6.26)	0.38 (0.08~1.91)	0.19 (0.04~1.07)
University—Bachelors		87.64% (475)	Reference	Reference	Reference	Reference	10.98% (18)	Reference	Reference	Reference	Reference
Father’s Education Level											
Senior high school and above	31.98 ***	42.62% (231)	2.21 * (1.09~4.47)	1.45 (0.82~2.56)	1.47 (0.86~2.51)	1.72 (0.98~3.00)	18.29% (30)	1.42 (0.40~5.10)	0.85 (0.22~3.35)	0.17 (0.02~1.33)	0.06 * (0.003~0.90)
Junior high school and below		57.38% (311)	Reference	Reference	Reference	Reference	81.71% (134)	Reference	Reference	Reference	Reference
Mother’s Education Level											
Senior high school and above	21.87 ***	33.58% (182)	0.62 (0.30~1.30)	0.53 * (0.28~0.98)	0.50 * (0.28~0.90)	0.98 (0.54~1.77)	14.63% (24)	0.49 (0.11~2.09)	0.92 (0.18~4.55)	1.55 (0.20~12.00)	2.84 (0.29~27.72)
Junior high school and below		66.42% (360)	Reference	Reference	Reference	Reference	85.37% (140)	Reference	Reference	Reference	Reference
Father’s Profession											
None or Individual Business	6.93 *	25.46% (138)	0.67 (0.30~1.47)	0.82 (0.43~1.57)	0.68 (0.37~1.26)	0.88 (0.46~1.65)	35.98% (59)	0.96 (0.36~2.52)	0.56 (0.18~1.72)	0.95 (0.27~3.35)	0.76 (0.19~2.95)
Farmer		32.29% (175)	0.80 (0.24~2.61)	0.81 (0.31~2.09)	0.98 (0.40~2.42)	1.44 (0.55~3.72)	28.04% (46)	0.22 * (0.05~0.98)	1.05 (0.25~4.45)	5.14 (0.96~27.63)	1.47 (0.21~10.54)
Worker or Employed		42.25% (229)	Reference	Reference	Reference	Reference	35.98% (59)	Reference	Reference	Reference	Reference
Mother’s Profession											
None or Individual Business	11.25 **	31.00% (168)	1.71 (0.79~3.71)	1.41 (0.74~2.68)	1.28 (0.70~2.34)	1.42 (0.76~2.65)	45.12% (74)	0.76 (0.29~2.00)	4.05 * (1.16~14.22)	1.22 (0.36~4.16)	1.43 (0.38~5.40)
Farmer		33.58% (182)	1.64 (0.47~5.70)	1.17 (0.44~3.12)	0.83 (0.33~2.12)	1.16 (0.43~3.14)	25.61% (42)	1.55 (0.33~7.19)	2.15 (0.39~11.73)	0.19 (0.03~1.27)	0.27 (0.03~2.52)
Worker or Employed		35.42% (192)	Reference	Reference	Reference	Reference	29.27% (48)	Reference	Reference	Reference	Reference
Living with Someone in Daily Life											
Father or mother	37.57 ***	10.33% (56)	0.96 (0.42~2.20)	1.54 (0.80~2.99)	1.38 (0.73~2.63)	0.90 (0.45~1.81)	26.22% (43)	1.45 (0.59~3.58)	0.68 (0.24~1.91)	1.26 (0.41~3.88)	0.85 (0.27~2.65)
Parents and grandparents		2.03% (11)	0.86 (0.10~7.56)	1.08 (0.21~5.67)	0.84 (0.16~4.37)	2.02 (0.52~7.81)	4.88% (8)	2.75 (0.51~14.8)	0.57 (0.06~5.34)	1.00 (0.09~11.27)	1.67 (0.15~18.71)
Other or Grandparents		6.46% (35)	0.80 (0.25~2.53)	1.71 (0.76~3.85)	1.67 (0.77~3.62)	0.79 (0.32~1.98)	10.37% (17)	2.35 (0.60~9.27)	0.83 (0.20~3.38)	5.39 * (1.27~22.81)	0.28 (0.03~2.83)
Parents		81.18% (440)	Reference	Reference	Reference	Reference	58.54% (96)	Reference	Reference	Reference	Reference
Contracting Systematic Disease											
Yes	0.00	2.95% (16)	1.24 (0.31~4.98)	0.69 (0.23~2.07)	0.93 (0.31~2.77)	0.57 (0.19~1.75)	3.05% (5)	0.08 (0.01~1.06)	0.61 (0.05~7.63)	0.98 (0.08~12.73)	0.17 (0.01~2.98)
No		97.05% (526)	Reference	Reference	Reference	Reference	96.95% (159)	Reference	Reference	Reference	Reference
Communicative Means											
Gesture and (or) lip-language or Word	592.64 ***	0 % (0)	None	None	None	None	87.20% (143)	0.23 * (0.06~0.85)	0.88 (0.23~3.39)	5.02 (0.47~54.18)	2.27 (0.27~18.86)
Mandarin		100% (542)	None	None	None	None	12.80% (21)	Reference	Reference	Reference	Reference
Satisfaction with the Current Communication Mode											
Satisfaction	87.28 ***	78.97% (428)	0.52 * (0.29~0.96)	0.59 * (0.35~0.97)	0.59 * (0.36~0.95)	0.61 (0.37~1.00)	40.85% (67)	0.20 *** (0.08~0.49)	2.01 (0.84~4.79)	1.59 (0.60~4.24)	1.13 (0.37~3.43)
Fair or Dissatisfaction		21.03% (114)	Reference	Reference	Reference	Reference	59.15% (97)	Reference	Reference	Reference	Reference
Exercise during the COVID~19 Pandemic											
Yes	0.22	78.78% (427)	0.65 (0.35~1.21)	0.70 (0.41~1.17)	0.84 (0.51~1.39)	1.47 (0.85~2.53)	80.49% (132)	1.58 (0.55~4.56)	1.95 (0.55~6.85)	1.41 (0.36~5.52)	1.69 (0.38~7.51)
No		21.22% (115)	Reference	Reference	Reference	Reference	19.51% (32)	Reference	Reference	Reference	Reference
Bedtime											
Before 9:00 pm	199.31 ***	2.95% (16)	0.72 (0.16~3.27)	0.25 (0.05~1.19)	0.23 * (0.06~0.99)	0.18 * (0.04~0.89)	26.83% (44)	2.20 (0.40~12.3)	0.36 (0.06~2.27)	4.13 (0.34~50.47)	0.77 (0.09~6.86)
9:00~10:00 pm		16.61% (90)	0.18 ** (0.06~0.56)	0.31 * (0.12~0.84)	0.24 ** (0.09~0.62)	0.18 ** (0.07~0.48)	48.78% (80)	2.03 (0.43~9.64)	0.42 (0.08~2.27)	2.36 (0.21~26.35)	0.38 (0.05~2.98)
10:00~11:00 pm		47.42% (257)	0.23 ** (0.09~0.60)	0.28 ** (0.11~0.67)	0.20 *** (0.09~0.47)	0.20 *** (0.08~0.46)	17.68% (29)	0.85 (0.15~4.91)	0.48 (0.08~3.06)	2.64 (0.20~34.32)	0.11 (0.01~1.33)
11:00~12:00 pm		26.38% (143)	0.46 (0.18~1.15)	0.54 (0.23~1.31)	0.43 * (0.19~0.99)	0.39 * (0.17~0.90)	6.71% (11)	Reference	Reference	Reference	Reference
After midnight		6.64% (36)	Reference	Reference	Reference	Reference	0% (0)	None	None	None	None

IES-R, the Impact of Events Scale-Revised questionnaire; OR, odds ratio; 95% CI, 95% confidence interval; * *p* < 0.05, ** *p* < 0.01, *** *p* < 0.001.

**Table 2:** Association of precautionary measures, concerns, and public service related to COVID-19 with the mental health status and psychological impact for students with normal hearing (*n* = 542) and students with hearing loss (*n* = 164).

Precautionary Measures, Concerns, and Public Service Variables	χ^2^	Students with Normal Hearing (*n* = 542)					Students with Hearing Loss (*n* = 164)				
		Percentage (*n*)	Stress	Anxiety	Depression	IES-R	Percentage (*n*)	Stress	Anxiety	Depression	IES-R
			OR (95% CI)	OR (95% CI)	OR (95% CI)	OR (95% CI)		OR (95% CI)	OR (95% CI)	OR (95% CI)	OR (95% CI)
Avoiding Sharing of Utensils (e.g., chopsticks) during Meals											
Most of the time	18.10 ***	24.35% (132)	0.60 (0.30~1.22)	0.95 (0.54~1.68)	0.97 (0.57~1.65)	1.23 (0.71~2.92)	19.51% (32)	3.54 * (1.21~10.35)	1.15 (0.36~3.64)	0.79 (0.22~2.88)	2.21 (0.55~8.88)
Sometime/ Occasionally/ Never		10.70% (161)	0.65 (0.33~1.28)	1.44 (0.66~1.97)	1.01 (0.60~1.69)	0.95 (0.54~1.66)	48.56% (78)	1.71 (0.68~4.28)	0.65 (0.24~1.74)	0.47 (0.15~1.46)	0.92 (0.28~4.48)
Always		45.94% (249)	Reference	Reference	Reference	Reference	32.93% (54)	Reference	Reference	Reference	Reference
Washing Hands with Soap and Water											
Most of the time	1.27	20.11% (109)	1.22 (0.61~2.44)	1.55 (0.89~2.72)	1.34 (0.79~2.27)	1.50 (0.86~2.60)	16.46% (27)	0.88 (0.22~3.48)	0.89 (0.24~3.35)	0.04 * (0.003~0.36)	0.30 (0.05~1.80)
Sometime/ Occasionally/ Never		10.52% (57)	2.17 (0.86~5.44)	2.34 * (1.08~5.08)	2.07 (0.99~4.32)	1.63 (0.75~3.54)	12.19% (20)	0.60 (0.18~2.00)	0.92 (0.19~4.41)	2.17 (0.38~12.42)	0.59 (0.08~4.51)
Always		69.37% (376)	Reference	Reference	Reference	Reference	71.34% (117)	Reference	Reference	Reference	Reference
Washing Hands Immediately after Coughing, Rubbing the Nose or Sneezing											
Most of the time	6.47 *	25.28% (137)	1.45 (0.73~2.91)	1.27 (0.71~2.26)	1.45 (0.84~2.48)	0.93 (0.52~1.65)	19.51% (32)	1.24 (0.41~3.79)	0.41 (0.11~1.44)	0.62 (0.14~2.80)	1.15 (0.27~4.95)
Sometime/ Occasionally/ Never		21.96% (119)	1.01 (0.43~2.34)	0.86 (0.43~1.72)	1.15 (0.61~2.18)	1.22 (0.63~2.35)	16.46% (27)	2.07 (0.63~6.76)	0.41 (0.10~1.72)	1.99 (0.48~8.17)	1.65 (0.26~10.71)
Always		52.77% (286)	Reference	Reference	Reference	Reference	64.02% (105)	Reference	Reference	Reference	Reference
Wearing Mask Regardless of the Presence or Absence of Symptoms											
Most of the time	6.22 *	23.06% (125)	0.90 (0.45~1.79)	1.00 (0.57~1.75)	1.12 (0.67~1.89)	1.19 (0.69~2.05)	14.02% (23)	6.12 ** (1.73~21.66)	1.65 (0.43~6.31)	6.19 * (1.17~32.66)	4.23 (0.77~23.21)
Sometime/ Occasionally/ Never		10.15% (55)	1.60 (0.66~3.92)	1.71 (0.82~3.59)	1.56 (0.77~3.17)	1.44 (0.69~3.03)	11.59% (19)	2.00 (0.49~8.23)	1.63 (0.32~8.32)	0.10 (0.006~1.58)	2.53 (0.36~17.64)
Always		66.79% (362)	Reference	Reference	Reference	Reference	74.39% (122)	Reference	Reference	Reference	Reference
Feeling Unnecessary Worry about the COVID-19 Outbreak											
Most of the time	10.55 **	19.19% (104)	0.67 (0.22~1.98)	0.69 (0.30~1.60)	0.79 (0.36~1.76)	0.81 (0.34~1.93)	15.85% (26)	0.90 (0.24~3.44)	1.71 (0.41~7.16)	0.62 (0.13~2.90)	1.56 (0.33~7.46)
Sometime/ Occasionally/ Never		69.38% (376)	0.96 (0.38~2.38)	0.70 (0.34~1.44)	0.77 (0.38~1.55)	0.91 (0.42~1.95)	62.81% (103)	1.48 (0.50~4.40)	2.11 (0.67~6.68)	0.73 (0.21~2.51)	0.45 (0.11~1.82)
Always		11.44% (62)	Reference	Reference	Reference	Reference	21.34% (35)	Reference	Reference	Reference	Reference
Number of Hours Staying at Home Per Day to Avoid COVID-19											
17~24 h	7.55 *	81.73% (443)	1.03 (0.32~3.34)	1.66 (0.59~4.69)	2.06 (0.74~5.74)	3.57 (0.98~12.99)	72.56% (119)	0.79 (0.24~2.61)	0.56 (0.15~2.08)	0.43 (0.09~2.10)	0.31 (0.07~1.46)
9~16 h		11.62% (63)	1.36 (0.36~5.14)	1.87 (0.58~6.02)	2.05 (0.65~6.48)	4.01 (0.99~16.23)	15.24% (25)	1.08 (0.25~4.63)	0.54 (0.10~2.79)	0.60 (0.09~3.84)	0.11 (0.01~1.41)
0~8 h		6.64% (36)	Reference	Reference	Reference	Reference	12.20% (20)	Reference	Reference	Reference	Reference
Recent Testing for COVID-19 in the Past 14 Days											
No	0.22	94.28% (511)	1.43 (0.37~5.52)	2.46 (0.68~8.84)	1.91 (0.61~5.99)	3.04 (0.82~11.33)	93.29% (153)	0.78 (0.17~3.68)	4.43 (0.44~44.27)	4.74 (0.36~63.28)	1.77 (0.17~18.51)
Yes		5.72% (31)	Reference	Reference	Reference	Reference	6.71% (11)	Reference	Reference	Reference	Reference
Concerned by Family Members Becoming Infected during the COVID-19 Outbreak in Beijing											
No	8.25 **	73.80% (400)	1.20 (0.64~2.27)	0.85 (0.50~1.46)	0.85 (0.51~1.40)	0.82 (0.49~1.37)	62.20% (102)	1.57 (0.24~1.36)	1.45 (0.55~3.84)	1.62 (0.55~4.75)	1.32 (0.37~4.67)
Yes		26.20% (142)	Reference	Reference	Reference	Reference	37.80% (62)	Reference	Reference	Reference	Reference
Concerned by New COVID-19 Outbreak in China during the Recent Confirmed Cases of COVID-19 Overseas											
No	0.58	42.99% (233)	0.53* (0.28~0.99)	0.91 (0.55~1.48)	0.87 (0.55~1.39)	0.74 (0.46~1.21)	39.63% (65)	0.71 (0.27~1.91)	1.19 (0.43~3.29)	0.69 (0.22~2.18)	1.30 (0.36~4.68)
Yes		57.01% (309)	Reference	Reference	Reference	Reference	60.37% (99)	Reference	Reference	Reference	Reference
The School has Educational Support during the COVID-19 Pandemic											
No	29.88 ***	9.59% (52)	1.32 (0.54~3.23)	1.32 (0.64~2.72)	1.16 (0.57~2.34)	0.98 (0.45~2.12)	26.22% (43)	1.15 (0.43~3.10)	0.96 (0.33~2.78)	1.94 (0.57~6.53)	0.98 (0.19~5.12)
Yes		90.41% (490)	Reference	Reference	Reference	Reference	73.78% (121)	Reference	Reference	Reference	Reference
The Community Provides Special Assistance during the COVID-19 Outbreak											
No	2.17	57.56% (312)	1.05 (0.55~2.01)	1.00 (0.58~1.72)	1.10 (0.66~1.82)	1.23 (0.72~2.09)	64.02% (105)	1.16 (0.43~3.12)	0.96 (0.33~2.78)	0.86 (0.26~2.91)	0.23 (0.05~1.00)
Yes		42.44% (230)	Reference	Reference	Reference	Reference	35.98% (59)	Reference	Reference	Reference	Reference
The Local Government Provides Special Attention and Support during the COVID-19 Pandemic											
No	25.62 ***	47.60% (258)	1.41 (0.74~2.67)	1.35 (0.80~2.29)	1.21 (0.74~1.98)	1.41 (0.84~2.36)	70.12% (115)	1.00 (0.39~2.53)	0.61 (0.23~1.64)	0.88 (0.27~2.88)	0.89 (0.22~3.60)
Yes		52.40% (284)	Reference	Reference	Reference	Reference	29.88% (49)	Reference	Reference	Reference	Reference
Needing Help during the COVID-19 Pandemic											
No	0.02	90.59% (491)	0.63 (0.29~1.37)	0.85 (0.41~1.76)	0.77 (0.40~1.52)	0.44 * (0.23~0.84)	90.24% (148)	0.67 (0.18~2.51)	0.63 (0.16~2.53)	2.35 (0.43~12.89)	0.76 (0.15~3.93)
Yes		9.41% (51)	Reference	Reference	Reference	Reference	9.76% (16)	Reference	Reference	Reference	Reference
Receiving Mental Health Counseling during the COVID-19 Pandemic											
No	1.58	86.53% (469)	0.87 (0.41~1.85)	0.89 (0.47~1.68)	1.07 (0.58~1.96)	0.63 (0.35~1.15)	90.24% (148)	1.43 (0.39~5.26)	1.40 (0.30~6.56)	0.10 ** (0.02~0.52)	0.24 (0.06~1.00)
Yes		13.47% (73)	Reference	Reference	Reference	Reference	9.76% (16)	Reference	Reference	Reference	Reference
Lifestyle Affected by COVID-19 Pandemic											
No	29.63 ***	49.82% (270)	0.43 ** (0.24~0.77)	0.62 * (0.39~0.99)	0.57 * (0.37~0.88)	0.46 ** (0.29~0.73)	73.78% (121)	0.39 * (0.17~0.92)	2.07 (0.73~5.94)	1.25 (0.40~3.92)	0.91 (0.25~3.27)
Yes		50.18% (272)	Reference	Reference	Reference	Reference	26.22% (43)	Reference	Reference	Reference	Reference

IES-R, the Impact of Events Scale-Revised questionnaire; OR, odds ratio; 95% CI, 95% confidence interval; * *p* < 0.05, ** *p* < 0.01, *** *p* < 0.001.

## Data Availability

The raw data supporting the conclusions of this article are available on request from the authors, without undue reservation.

## Appendix A

**Table A1:** The relation between psychological inflexibility and the psychological impact as well as mental health status for students with hearing loss (*n* = 164).

Item of Psychological Inflexibility for the Student with Hearing Loss	Percentage (*n*)	Stress		Anxiety		Depression		IES-R	
		Mean (SD)	T	Mean (SD)	T	Mean (SD)	T	Mean (SD)	T
I am Leading a Full Life, Despite My Frustration with Hearing Loss									
Always	26.22% (43)	5.26 8.56)	−0.20	5.12 (6.36)	0.79	4.23 (6.81)	0.61	9.28 (12.26)	−0.45
Most of the time	26.22% (43)	10.28 (8.53)	2.11 *	3.86 (5.97)	0.04	4.70 (7.02)	0.86	10.53 11.81)	−0.05
Sometime	26.22% (43)	10.88 (7.86)	2.54 *	2.60 (4.87)	−0.85	4.33 (8.26)	0.57	11.37 (16.01)	0.18
Occasionally	9.76% (16)	10.00 (6.69)	1.97	3.75 (6.40)	−0.02	6.00 (9.32)	1.14	16.25 (19.62)	1.05
Never	11.59% (19)	5.68 (6.26)	Ref.	3.79 (5.49)	Ref.	3.16 (5.18)	Ref.	10.68 (8.85)	Ref.
My Life is Going Well, Despite Negative Thoughts and Feelings about My Hearing Loss									
Always	26.22% (43)	4.33 (7.02)	−1.18	4.47 (6.09)	0.63	3.77 (6.30)	−0.40	9.63 (11.44)	−0.75
Most of the time	33.54% (55)	10.04 (9.36)	1.24	4.04 (5.68)	0.42	4.25 (6.81)	−0.14	9.49 (12.26)	−0.77
Sometime	21.95% (36)	9.39 (6.57)	1.26	3.89 (6.59)	0.28	4.06 (7.85)	−0.21	11.44 (15.27)	−0.17
Occasionally	9.15% (15)	15.07 (6.13)	3.44 **	1.73 (2.81)	−0.96	7.73 (10.85)	0.98	18.20 (20.70)	0.98
Never	9.15% (15)	6.80 (7.00)	Ref.	3.33 (5.79)	Ref.	4.53 (6.44)	Ref.	12.20 (11.68)	Ref.
My Frustration with Hearing Loss has Made Me Less Involved in Activities I Enjoy									
Always	6.71% (11)	10.00 (11.97)	1.48	6.36 (7.15)	1.20	0.36 (0.81)	−3.96 ***	5.27 (6.23)	−1.30
Most of the time	14.02% (23)	11.30 (9.28)	3.21 **	4.87 (7.11)	0.58	5.74 (8.49)	1.07	15.43 (16.84)	1.56
Sometime	29.27% (48)	10.29 (7.26)	4.26 ***	2.75 (5.03)	−1.10	5.13 (7.86)	1.05	10.96 (14.34)	0.57
Occasionally	20.73% (34)	9.53 (8.64)	3.11 **	3.71 (5.44)	−0.20	4.94 (8.76)	0.81	12.03 (15.98)	0.81
Never	29.27% (48)	4.50 (6.00)	Ref.	3.96 (5.74)	Ref.	3.67 (5.52)	Ref.	9.50 (10.34)	Ref.
I Wish I Could Control Negative Thoughts and Feelings about My Hearing Loss									
Always	14.63% (24)	7.58 (7.93)	1.33	5.17 (6.21)	0.44	5.00 (7.17)	0.50	14.25 (14.60)	1.66
Most of the time	15.85% (26)	9.38 (10.10)	2.08 *	5.62 (6.97)	0.71	6.62 (9.94)	1.13	13.58 (17.02)	1.33
Sometime	26.83% (44)	9.95 (7.94)	3.02 **	2.32 (3.86)	−1.97	3.50 (5.93)	−0.54	9.14 (10.43)	0.19
Occasionally	17.07% (28)	11.64 (7.58)	3.73	2.50 (5.98)	−1.35	3.71 (8.57)	−0.28	12.14 (18.63)	0.89
Never	25.61% (42)	5.10 (6.93)	Ref.	4.48 (6.02)	Ref.	4.19 (5.86)	Ref.	8.74 (9.47)	Ref.
Frustration with Hearing Loss does not Interfere with My Goals									
Always	26.22% (43)	3.86 (6.39)	−1.40	4.37 (5.91)	−0.63	4.19 (6.14)	−0.55	9.47 (11.12)	−1.07
Most of the time	18.29% (30)	9.07 (8.96)	1.30	4.27 (5.70)	−0.64	3.87 (6.58)	−0.64	8.20 (11.60)	−1.35
Sometime	29.27% (48)	12.00 (7.31)	3.23	3.08 (5.47)	−1.29	3.96 (6.18)	−0.69	11.19 (13.72)	−0.44
Occasionally	12.80% (21)	12.10 (9.60)	2.41 *	2.19 (3.46)	−1.79	6.00 (11.31)	0.27	15.95 (20.95)	0.63
Never	13.41% (22)	6.18 (6.23)	Ref.	5.45 (7.79)	Ref.	5.18 (8.34)	Ref.	12.68 (12.07)	Ref.
Despite Negative Thoughts and Feelings about My Hearing Loss, I Can Still Take Care of My Responsibilities									
Always	32.93% (54)	5.26 (7.26)	−0.99	4.22 (5.99)	0.60	5.04 (8.41)	0.39	11.26 (13.98)	0.32
Most of the time	32.93% (54)	11.48 (8.83)	1.78	4.93 (6.45)	1.15	4.00 (7.31)	−0.06	10.37 (14.40)	0.09
Sometime	18.29% (30)	10.53 (8.10)	1.40	2.27 (5.09)	−0.64	3.33 (4.53)	−0.45	10.17 (11.50)	0.04
Occasionally	6.10% (10)	6.80 (7.07)	−0.17	1.60 (3.37)	−0.97	7.20 (8.01)	0.99	17.10 (15.88)	1.21
Never	9.76% (16)	7.25 (6.44)	Ref.	3.25 (4.67)	Ref.	4.13 (7.50)	Ref.	10.00 (13.71)	Ref.
I Struggle to Get Things Done Because of My Frustration with Hearing Loss									
Always	4.88% (8)	12.00 (13.27)	1.63	10.00 (7.48)	2.21	5.25 (8.94)	0.54	5.88 (10.40)	−0.93
Most of the time	7.93% (13)	14.92 (10.44)	4.60 ***	3.38 (4.72)	−0.33	7.08 (10.73)	1.04	17.54 (22.17)	1.58
Sometime	34.15% (56)	10.43 (7.22)	4.54 ***	3.36 (5.87)	−0.49	4.68 (7.53)	0.60	12.77 (14.03)	0.99
Occasionally	26.22% (43)	8.05 (7.46)	2.61 *	3.40 (5.90)	−0.43	3.77 (6.40)	−0.04	8.56 (11.63)	−0.62
Never	26.83% (44)	4.18 (6.29)	Ref.	3.91 (5.16)	Ref.	3.82 (6.51)	Ref.	10.14 (12.10)	Ref.
I Need to Manage Negative Thoughts About My Hearing Loss to Have Control Over My Life									
Always	7.32% (12)	7.33 (8.11)	1.45	6.50 (7.29)	0.98	3.33 (4.46)	−0.36	10.58 (12.73)	0.31
Most of the time	12.20% (20)	9.20 (10.23)	1.99	6.20 (7.51)	1.02	5.80 (7.81)	0.96	12.40 (15.90)	0.76
Sometime	28.66% (47)	11.15 (8.29)	4.60 ***	2.77 (4.50)	−1.44	4.21 (6.45)	0.13	10.26 (10.57)	0.37
Occasionally	22.56% (37)	10.81 (7.84)	4.34 ***	2.49 (5.36)	−1.48	4.81 (9.70)	0.44	13.38 (18.63)	1.14
Never	29.27% (48)	4.33 (5.94)	Ref.	4.29 (5.72)	Ref.	4.04 (6.52)	Ref.	9.42 (11.37)	Ref.
My Negative Thoughts and Feelings About My Hearing Loss Lead Me to Avoid Situations									
Always	5.49% (9)	6.00 (7.62)	0.43	3.78 (4.41)	−0.33	3.72 (5.87)	0.93	16.44 (15.37)	2.10 *
Most of the time	7.93% (13)	9.38 (6.85)	1.99	3.23 (6.03)	−0.67	8.00 (11.17)	1.34	17.38 (21.71)	1.51
Sometime	36.59% (60)	11.50 (9.10)	4.15 ***	3.63 (5.36)	−0.78	3.67 (6.02)	−0.05	10.57 (11.78)	1.20
Occasionally	19.51% (32)	9.25 (6.26)	2.78 **	3.50 (6.70)	−0.69	4.88 (8.91)	0.71	12.34 (16.63)	1.32
Never	30.49% (50)	4.84 (7.43)	Ref.	4.48 (6.03)	Ref.	3.72 (5.87)	Ref.	8.02 (10.09)	Ref.
I Worry About What Others Think of My Hearing Loss									
Always	4.27% (7)	13.71 (13.34)	1.63	8.29 (7.06)	1.40	3.43 (5.13)	−0.24	6.71 (10.93)	−0.82
Most of the time	10.37% (17)	10.94 (7.04)	2.94 **	5.06 (7.28)	0.19	5.18 (7.25)	0.62	9.65 (13.96)	−0.27
Sometime	25.00% (41)	9.90 (7.51)	3.12 **	3.37 (5.54)	−1.11	4.83 (7.94)	0.55	13.24 (15.11)	0.98
Occasionally	25.00% (41)	9.85 (9.16)	2.79 **	1.80 (3.49)	−2.96 **	4.44 (8.38)	0.27	10.66 (15.21)	0.03
Never	35.37% (58)	5.38 (6.81)	Ref.	4.72 (6.27)	Ref.	4.03 (6.46)	Ref.	10.57 (11.91)	Ref.
I Spend a Lot of Time Thinking about How Things Would Be for Me without Hearing Loss									
Always	7.32% (12)	11.00 (11.62)	1.84	7.33 (6.95)	1.24	1.67 (3.17)	−1.70	7.58 (9.93)	−1.05
Most of the time	8.54% (14)	12.00 (8.38)	3.40 **	3.43 (5.52)	−0.66	4.14 (6.99)	0.09	7.50 (13.63)	−1.03
Sometime	35.37% (58)	9.21 (6.91)	3.39 **	3.07 (5.48)	−1.34	5.62 (8.55)	1.07	14.57 (17.19)	1.37
Occasionally	22.56% (37)	10.11 (9.28)	3.10 **	3.08 (4.96)	−1.25	4.11 (7.21)	0.10	7.97 (11.91)	−1.21
Never	26.22% (43)	4.56 (6.67)	Ref.	4.70 (6.41)	Ref.	3.95 (6.48)	Ref.	10.88 (9.58)	Ref.
Frustration with My Hearing Loss Keeps Me from Effectively Treating and Managing It									
Always	4.27% (7)	14.86 (12.59)	2.20	6.86 (7.65)	1.29	2.29 (5.22)	−0.72	8.00 (11.31)	−0.41
Most of the time	7.32% (12)	8.50 (7.09)	2.17 *	5.83 (6.58)	1.09	7.67 (11.18)	1.33	15.67 (22.76)	0.87
Sometime	22.56% (37)	11.03 (8.23)	4.71 ***	3.62 (5.79)	−0.18	3.73 (6.02)	−0.42	11.00 (13.31)	0.47
Occasionally	28.05% (46)	11.43 (8.08)	5.29 ***	3.04 (5.46)	−0.73	4.61 (7.48)	0.20	11.83 (14.63)	0.80
Never	37.80% (62)	4.26 (6.02)	Ref.	3.84 (5.68)	Ref.	4.32 (7.24)	Ref.	9.82 (11.29)	Ref.

IES-R, the Impact of Events Scale-Revised questionnaire; Ref. = Reference; * *p* < 0.05, ** *p* < 0.01, *** *p* < 0.001.

**Table A2:** Varimax Rotated Factor Structure of the Acceptance and Action Questionnaire—Adult Hearing Loss (AAQ-AHL).

Item	AAQ-AHL	Factor 1	Factor 2
01	I am leading a full life, despite my frustration with hearing loss	−0.032	0.835
02	My life is going well, despite negative thoughts and feelings about my hearing loss	0.079	0.887
03	My frustration with hearing loss has made me less involved in activities I enjoy	0.740	0.170
04	I wish I could control negative thoughts and feelings about my hearing loss	0.644	−0.149
05	Frustration with hearing loss does not interfere with my goals	0.090	0.832
06	Despite negative thoughts and feelings about my hearing loss, I can still take care of my responsibilities	−0.025	0.803
07	I struggle to get things done because of my frustration with hearing loss	0.774	0.314
08	I need to manage negative thoughts about my hearing loss to have control over my life	0.772	0.056
09	My negative thoughts and feelings about my hearing loss lead me to avoid situations	0.786	−0.048
10	I worry about what others think of my hearing loss	0.764	−0.021
11	I spend a lot of time thinking how things would be for me without hearing loss	0.766	−0.054
12	Frustration with my hearing loss keeps me from effectively treating and managing it	0.835	0.087

The highest loading obtained by a variable among the factor is underlined.

**Table A3:** Item-total Correlations and Reliability Measures of Factor Structure of AAQ-AHL.

Factor	Item	CITC	Cronbach α CADT	Cronbach α CF
Factor 1	Item 03	0.662	0.881	0.894
	Item 04	0.543	0.895	
	Item0 7	0.706	0.878	
	Item 08	0.696	0.878	
	Item 09	0.712	0.877	
	Item 10	0.665	0.881	
	Item 11	0.673	0.880	
	Item 12	0.759	0.873	
Factor 2	Item 01	0.715	0.828	0.865
	Item 02	0.779	0.803	
	Item 05	0.709	0.831	
	Item 06	0.660	0.849	

CITC: Corrected items total correlation; Cronbach α CADT: Cronbach α coefficient after deleting terms; Cronbach α CF: Cronbach α coefficient of factor.
